# Ambulatory low cardiac output state in heart failure: characteristics and outcomes

**DOI:** 10.1093/eschf/xvag215

**Published:** 2026-08-19

**Authors:** Benjamin Lautrup Hansen, Tania Deis, Cecilie Müller Ralsted, Kasper Rossing, Mads Ersbøll, Hoong Sern Lim, Finn Gustafsson

**Affiliations:** Department of Cardiology, Rigshospitalet, Copenhagen University Hospital, Blegdamsvej 9, Copenhagen OE 2100, Denmark; Department of Cardiology, Rigshospitalet, Copenhagen University Hospital, Blegdamsvej 9, Copenhagen OE 2100, Denmark; Department of Cardiology, Rigshospitalet, Copenhagen University Hospital, Blegdamsvej 9, Copenhagen OE 2100, Denmark; Department of Cardiology, Rigshospitalet, Copenhagen University Hospital, Blegdamsvej 9, Copenhagen OE 2100, Denmark; Department of Cardiology, Rigshospitalet, Copenhagen University Hospital, Blegdamsvej 9, Copenhagen OE 2100, Denmark; Department of Cardiology, University Hospitals Birmingham NHS Foundation Trust, Birmingham, UK; Institute of Cardiovascular Sciences, University of Birmingham, Birmingham, UK; Department of Cardiology, Rigshospitalet, Copenhagen University Hospital, Blegdamsvej 9, Copenhagen OE 2100, Denmark

**Keywords:** Cardiac index, Cardiac output, Heart failure with reduced ejection fraction, Invasive hemodynamics, Right heart catheterization

## Abstract

**Introduction:**

Ambulatory low cardiac output state (LCOS) is a known but poorly described heart failure (HF) phenotype. We aimed to investigate the prevalence, clinical markers, and prognostic importance of ambulatory LCOS.

**Methods:**

Consecutive ambulatory patients with HF and reduced left ventricular ejection fraction referred for advanced HF assessment were included. All patients underwent right heart catheterization. Patients with LCOS defined as a cardiac index <1.8 L/min/m^2^ were compared to patients without LCOS.

**Results:**

In total, 618 patients were included. The mean ± SD age was 52.5 ± 12.5 years, and 78% were male. Low cardiac output state was present in 116 patients (19% [95% CI 16 to 22%]). Patients with LCOS presented with more advanced symptoms, lower systolic blood pressure and LVEF, higher N-terminal pro-B-type natriuretic peptide levels, and were less likely to receive treatment with renin-angiotensin system inhibitors (RASi) compared with patients without LCOS. Absence of RASi therapy was strongly associated with LCOS, odds ratio 2.34 [95% CI 1.44 to 3.74], *P* < .001. While liver function markers were more elevated in patients with LCOS, renal function was similar between groups. Over a median follow-up of five years, LCOS was independently associated with mortality, hazard ratio 1.86 [95% 1.32 to 2.62], *P* < .001 (adjusted for age, sex, and advanced therapy).

**Conclusion:**

LCOS was observed in 19% of ambulatory patients and was strongly associated with mortality, independent of left-sided filling pressures and advanced therapies. Among other factors, the absence of RASi therapy was highly predictive of LCOS.

## Graphical Abstract

**Graphical Abstract:**
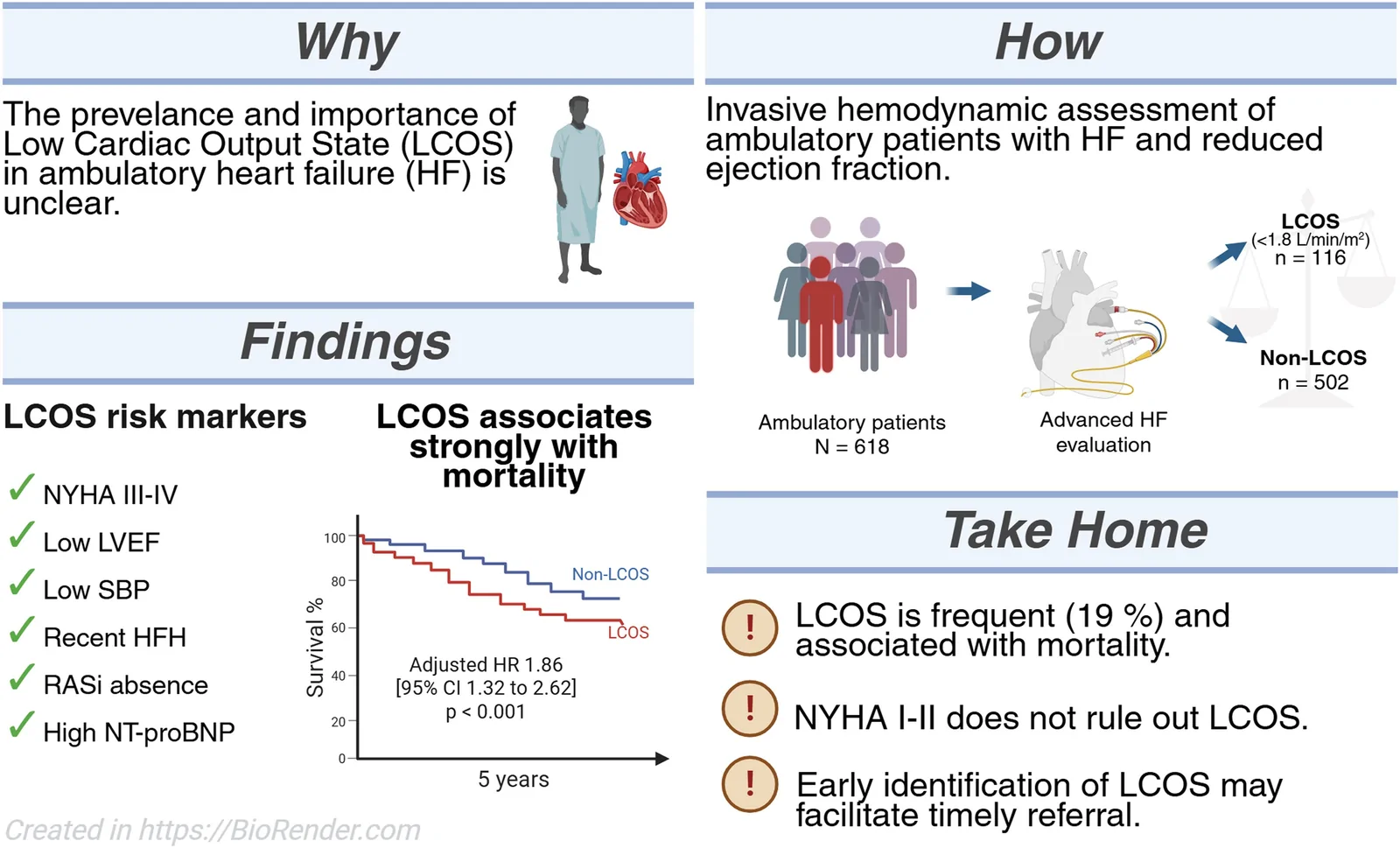
Ambulatory LCOS is observed in 19% of patients with HFrEF referred for evaluation for advanced therapies. Risk markers can assist clinicians in discriminating LCOS from advanced HF in general. However, patients with LCOS suffer an ominous prognosis despite utilization of advanced therapies.

## Introduction

Advanced heart failure (HF) is defined as persisting, severe symptoms despite optimal guideline-directed therapy.^1,2^ Recurrent hospitalizations are included in the definition of advanced HF, with overt congestion representing the most common cause of HF hospitalization (HFH).^3^ Severe congestion driven by elevated cardiac filling pressures, often leading to hospitalization, is a clear clinical sign and hallmark of worsening HF. Hypoperfusion, the other important component of advancing HF, is well recognized in patients with cardiogenic shock (CS) but less often considered in ambulatory patients. However, outpatients with chronic advanced HF may progressively decline from a congested state with relatively preserved cardiac output (CO) to a low cardiac output state (LCOS).^4^ Accurate diagnosis of LCOS requires invasive right heart catheterization (RHC), in part explaining the less frequent clinical suspicion of the syndrome.^5^ Haemodynamic criteria for LCOS in the setting of CS include a cardiac index <1.8 L/min/m^2^, often in combination with increased pulmonary capillary wedge pressure (PCWP) of ≥15 mmHg.^6^ According to the Society for Cardiovascular Angiography and Interventions (SCAI) classification system, a low cardiac index in the setting of shock translates into stage C ‘Classic CS’, which has been investigated in several studies.^6–10^ However, unlike patients in shock, ambulatory individuals with LCOS, sometimes referred to as ‘walking shock’, typically maintain normal lactate levels at rest and appear haemodynamically stable, even though end-organ dysfunction may already be developing. Consequently, LCOS may remain clinically unrecognized in ambulatory patients with chronic HF. Early identification is essential for timely referral for advanced therapies such as durable left ventricular assist device (LVAD) or heart transplant (HTx) listing. Very limited information about ambulatory LCOS from a non-contemporary cohort is available, and clinical markers of LCOS have not been reported.^11^ The aim of this study was to characterize LCOS in a large cohort of ambulatory patients with HF with reduced left ventricular ejection fraction (HFrEF) referred for invasive evaluation at a tertiary advanced HF centre. First, we explored clinical and pathophysiological differences between patients with LCOS and relatively preserved output HF (non-LCOS). Second, we investigated clinical markers of LCOS. Finally, we investigated the prognostic importance of LCOS and the association between filling pressures and clinical outcomes in patients with LCOS.

## Methods

### Patients and setting

At our tertiary advanced HF centre, we included consecutive elective patients with HFrEF referred for RHC as part of work-up for suspected advanced HF. Data and methods are described in previously published work,^12^ but the database was recently updated to contain 726 patients referred to our institution from January 2002 until October 2023. In brief, all patients underwent RHC per clinical indication as part of the evaluation for candidacy for HTx or LVAD. All RHC procedures were performed in the cardiac catheterization laboratory, and patients were admitted to the HF ward, not the cardiac intensive care unit (ICU). Most patients had severe HF symptoms at the time of RHC, but those in New York Heart Association (NYHA) class II were also evaluated invasively if they had recently experienced more severe symptoms or exhibited other high-risk features. For the purpose of present study, we excluded patients with a left ventricular ejection fraction (LVEF) >40% (*n* = 16), patients treated intravenously with inotropes (*n* = 38), and those with unavailable data for CO (*n* = 54). No patient had a temporary mechanical ventricular assist device during RHC. Thus, in summary, the study cohort represents ambulatory patients with HFrEF treated outside of the ICU. The study conforms with the principles outlined in the Declaration of Helsinki and was approved by the Danish Medical Agency (3-3013-1365/1) and the Data Protection Agency (RH 2015-153). Individual patient consent was not required for this observational study.

### Data

Data from the routine clinical work-up and RHC measures were collected from medical records, retrospectively. Blood biochemistry data was recorded in the closest proximity to the RHC procedure but at least within one week prior to the RHC procedure. Right heart catheterization procedures were performed with a balloon-tipped, 7F Swan-Ganz catheter inserted via the internal jugular or femoral vein. Guided by the pressure tracing on the monitor, the catheter was placed in the pulmonary artery, and pressure measurements were obtained simultaneously. Cardiac output was measured in triplicate using thermodilution with cold isotonic solutions of saline or glucose. Du Bois method was used to calculate body surface area in square metres (BSA = 0.007184 × height_cm_^0.725^ × weight_kg_^0.425^). Low cardiac output state was defined as a cardiac index <1.8 L/min/m^2^.

### Statistical analysis

Data are presented as mean ± SD or median (p25, p75), depending on the distribution. Comparisons for categorical variables were analysed using the Fisher exact test, while continuous variables were compared using the Mann–Whitney *U* test. Cardiac output and cardiac index were assessed both as continuous and grouped variables. An exact binomial test was performed to get 95% confidence limits on the prevalence of LCOS. With logistic regression models adjusted for age and sex, we computed odds ratios to assess associations between different explanatory variables and the presence of LCOS (yes/no). Patients were followed from the time of index RHC until 1 July 2025, and were censored if death, LVAD, or heart transplant had not occurred at the end of follow-up. Follow-up was capped at 5 years, and individuals with longer follow-up were censored at that time. Survival plots were created using Kaplan–Meier and Aalen–Johansen estimator curves, and risk estimates were computed with Cox and Fine–Gray regression. Model assumptions for regression analyses were validated graphically and statistically. In case of missing data, analyses were performed on complete cases only. A 2-sided *P*-value of <.05 was considered significant. R software (version 2024.4.2.764) was used.

## Results

In total, 618 patients were included. Overall population mean and median age were 52.5 ± 12.5 and 54 (45, 62) years (range 16–77 years), respectively. Seventy-eight per cent were male. Overall mean and median cardiac index were 2.41 ± 0.70 and 2.33 (1.92, 2.80) L/min/m^2^ (range 1.0–6.1 L/min/m^2^), respectively. Low cardiac output state was present in 116 patients (18.8% [95% CI 15.8 to 22.1]).

### Comparing clinical characteristics in patients LCOS and non-LCOS

Patients with LCOS were slightly younger than patients with relatively preserved resting cardiac output (*Table 1*). No significant differences were observed for sex and HF aetiology (ischaemic vs non-ischaemic). Mean systolic blood pressure (SBP) was significantly lower in the LCOS group, but the proportion of hypotensive patients (i.e. SBP <90 mmHg) was similar between groups. The prevalence of NYHA class III-IV symptoms was significantly higher in the LCOS group, but 28% of patients with LCOS reported mild to moderate HF symptoms (NYHA II). Similar rates of diabetes, obesity, atrial fibrillation, and chronic obstructive pulmonary disease were observed for the two groups. Duration of HF was comparable in both groups. A prior history of HFH was similar between the LCOS and non-LCOS groups, but recent HFH within six months was significantly more frequent among patients with LCOS. Patients with LCOS were more likely to have a cardiac resynchronization device and less likely to have an implantable cardioverter defibrillator, although these differences were not statistically significant. Low cardiac output state was associated with a significantly lower median LVEF (15 vs 20%) and a significantly higher mean heart rate (83 vs 78 bpm) compared with the non-LCOS group. Thus, despite a similar left ventricular end-diastolic diameter, the lower cardiac index in the LCOS group was largely attributable to a markedly reduced stroke volume (38 ± 11 mL and 71 ± 26 mL, *P* < .001). Both groups were relatively well treated with guideline-directed medical therapy. Seventy-two per cent of patients with LCOS were treated with a beta-blocker compared to 73% in the non-LCOS group. Use of a mineralocorticoid receptor antagonist was similar between groups. Renin-angiotensin system inhibitors (RASi) were prescribed in 71% of LCOS patients vs 85% of non-LCOS patients. Use of sodium-glucose cotransporter-2 inhibitors was comparably infrequent in both groups. Blood biochemistry showed no difference regarding levels of haemoglobin, sodium, potassium, and creatinine or estimated glomerular filtration rate (eGFR). Slightly more elevated levels of alkaline phosphatase (ALP), alanine aminotransferase (ALT), and bilirubin were observed in those with LCOS, although median values were all within normal reference intervals. The median N-terminal pro-B-type natriuretic peptide (NT-proBNP) was significantly more elevated in patients with LCOS compared to patients without (4533 vs 1877 pg/mL). As for invasive haemodynamics, compared to normal physiological values, we observed an elevated central venous pressure (CVP), mean pulmonary artery (PA) pressure, and pulmonary capillary wedge pressure (PCWP) for both groups. In the LCOS group, elevation of central haemodynamic pressures was more pronounced and pulmonary vascular resistance (PVR) was significantly higher. Mean pulmonary artery oxygen saturation of mixed venous blood was low in both groups but significantly lower by 9% points in patients with LCOS (54 vs 63%). Baseline characteristics are summarized in *Table 1*.

**Table 1:** Characteristics at baseline right heart catheterization

	LCOS N = 116	Non-LCOS N = 502	*P*-value*
Age, years	50.5 ± 14.3	52.9 ± 12.0	.088
Sex, male	91 (78%)	389 (77%)	.902
BMI, kg/m^2^	26.2 ± 5.11	26.9 ± 5.07	.141
Body surface area, m^2^	1.97 ± 0.24	2.01 ± 0.24	.160
Systolic blood pressure, mmHg (*n* = 500)	100 ± 13.8	105 ± 17.6	**<.001**
Systolic blood pressure <90 mmHg	21 (23%)	75 (18%)	.382
Mean arterial pressure (*n* = 497)	76 ± 10.6	79 ± 12.3	.**010**
Heart rate, bpm (*n* = 509)	83 ± 20	78 ± 17	.**027**
NYHA class III-IV (*n* = 583)	78 (72%)	264 (56%)	.**002**
Duration of HF, years	2.0 (0.0, 6.0)	2.0 (0.0, 8.0)	.154
Age at HF diagnosis	47 ± 13.3	48 ± 11.7	.230
Previous HFH (*n* = 611)	89 (79%)	378 (76%)	.461
Recent HFH, within 6 months	75 (67%)	248 (50%)	.**001**
Etiology			
Ischaemic	34 (29%)	164 (33%)	
Non-ischaemic^a^	82 (71%)	338 (67%)	.510
Device therapy			
Total ICD (ICD or CRT-D)	40 (34%)	184 (37%)	.748
Total CRT (CRT-P or CRT-D)	28 (24%)	86 (17%)	.085
Brady pacemaker	4 (3.4%)	13 (2.6%)	.540
Comorbidities			
Diabetes	18 (16%)	93 (19%)	.504
BMI ≥27 kg/m^2^	42 (36%)	212 (42%)	.251
Atrial fibrillation or flutter	43 (37%)	176 (35%)	.747
COPD	4 (3.4%)	38 (7.6%)	.150
Medication (*n* = 612)			
Beta-blocker	83 (72%)	364 (73%)	.816
RASi	82 (71%)	424 (85%)	**<.001**
MRA	64 (56%)	308 (62%)	.244
SGLT2i	10 (8.7%)	36 (7.2%)	.560
Loop diuretic	104 (90%)	442 (89%)	.740
Paraclinical characteristics			
Haemoglobin, mmol/L (*n* = 537)	8.6 ± 1.25	8.5 ± 1.15	.359
Sodium (*n* = 541)	137 ± 4.38	138 ± 3.94	.296
Potassium (*n* = 541)	4.0 ± 0.47	4.1 ± 0.44	.294
Creatinine, mg/dL (*n* = 553)	1.11 (0.91, 1.44)	1.13 (0.91, 1.46)	.618
eGFR, mL/min/1.73 m^2^ (*n* = 553)^b^			
Under 30	3 (3.0%)	15 (3.3%)	
30 to 60	32 (32%)	133 (29%)	
Above 60	65 (65%)	305 (67%)	.909
Alanine aminotransferase, U/L (*n* = 471)	32 (24, 60)	28 (20, 45)	.**009**
Alanine aminotransferase >3 × ULN^c^	3 (2.6%)	11 (2.2%)	.734
Alkaline phosphatase (*n* = 461)	93 (71, 131)	82 (63, 113)	.**025**
Alkaline phosphatase >3×ULN^d^	0 (0%)	3 (0.6%)	.095
Bilirubin, µmol/L (*n* = 461)	18 (11, 30)	13 (8, 20)	**<.001**
Bilirubin >ULN^e^	23 (20%)	41 (8.2%)	**<.001**
N-terminal pro-B-type natriuretic peptide, pg/mL (*n* = 209)	4533 (2469, 7163)	1877 (871, 4448)	**<.001**
Left ventricular ejection fraction, %	15 (10, 20)	20 (15, 25)	.**004**
Left ventricular end-diastolic diameter, mm (*n* = 583)	67 (58, 74)	67 (60, 73)	.358
TAPSE, mm (*n* = 388)	14 ± 4.9	17 ± 5.2	**<.001**
Right heart catheterization			
CVP, mmHg (*n* = 607)	13.3 ± 5.86	9.7 ± 5.10	**<.001**
CVP ≤8 mmHg	28 (25%)	257 (52%)	**<.001**
Systolic PAP (*n* = 610)	46 ± 15.2	41 ± 15.3	.**002**
Diastolic PAP (*n* = 610)	24 ± 7.7	20 ± 8.1	**<.001**
Mean PAP (*n* = 615)	32 ± 9.8	28 ± 10.4	**<.001**
PCWP (*n* = 616)	23 ± 7.7	19 ± 8.1	**<.001**
PCWP ≤14 mmHg	19 (16%)	179 (36%)	**<.001**
Cardiac Output, L/min	3.07 ± 0.55	5.24 ± 1.42	
Stroke Volume, mL (*n* = 509)	38 ± 11	71 ± 26	**<.001**
Cardiac Index, L/min/m^2^	1.55 ± 0.18	2.61 ± 0.62	
PVR, Wood unit (*n* = 617)	2.57 (1.79, 3.62)	1.62 (1.07, 2.34)	**<.001**
SvO_2_, % (*n* = 437)	54 ± 9.2	63 ± 9.3	**<.001**
Cardiac power output index, Watt/m^2^ (*n* = 491)^f^	0.21 ± 0.05	0.41 ± 0.14	**<.001**

Bold indicates a *P*-value <.05.

BMI, body mass index; COPD, chronic obstructive pulmonary disease; CPO, cardiac power output; CRT, cardiac resynchronization therapy; CVP, central venous pressure; eGFR, estimated glomerular filtration rate; HF, heart failure; HFH, heart failure hospitalization; ICD, implantable cardioverter defibrillator; MRA, mineralocorticoid receptor antagonist; NYHA, New York Heart Association class; PAP, pulmonary artery pressure; PCWP, pulmonary capillary wedge pressure; PVR, pulmonary vascular resistance; RASi, renin–angiotensin system inhibitor; SGLT2i, sodium-glucose cotransporter-2 inhibitor; SpO_2_, peripheral oxygen saturation; SvO_2_, mixed venous oxygen saturation; TAPSE, tricuspid annular plane systolic excursion.

^*^Mann–Whitney *U* test and 2-sample *t*-test were used for continuous variables. Fisher’s exact test was used for count data.

^a^All other aetiologies than ischaemic.

^b^CKD-EPI Creatinine Equation (2021).

^c^Upper limit normal defined as 70 U/L (males) and 45 U/L (females).

^d^Upper limit normal defined as 105 U/L for both sexes.

^e^Upper limit normal defined as 25 µmol/L.

^f^CPO = (mean arterial pressure—central venous pressure) × cardiac output/451/body surface area.

### Factors associated with LCOS

**Figure 1:**
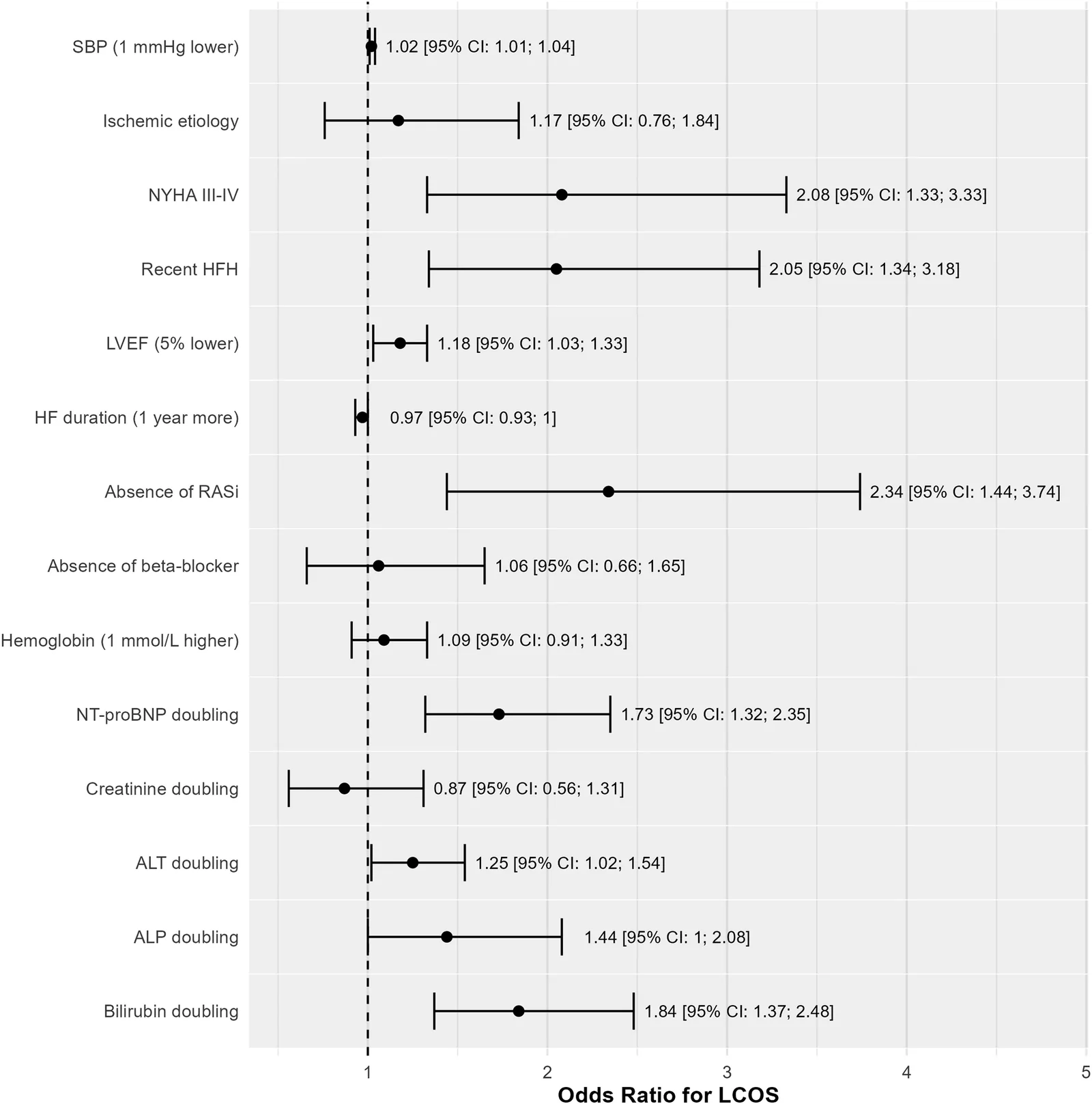
Clinical markers of ambulatory LCOS. Unadjusted odds ratios are provided. ALT, alanine aminotransferase; ALP, alkaline phosphatase

In unadjusted logistic regression models, no association was observed with age, sex, duration of HF, ischaemic aetiology, haemoglobin, and creatinine. Systolic blood pressure, NYHA class III-IV, recent HFH, and LVEF were all significantly associated with LCOS. Absence of RASi therapy was associated with an increased risk of LCOS (OR 2.34 [95% CI 1.44 to 3.74], *P* < .001), but absence of beta-blocker therapy was not. Natriuretic peptide was strongly associated with LCOS (OR for a doubling of NT-proBNP 1.73 [95% CI 1.32 to 2.35], *P* < .001). A doubling of liver function markers such as ALP, ALT, and bilirubin was also significantly associated with LCOS. Odds ratios are summarized in *Figure 1*. Adjustments for age and sex did not significantly change the results, except for ALT, which lost its statistical significance (*P* > .05). When exploring the effect of adjustment for CVP in the liver function analyses, both ALP and bilirubin lost, but ALT regained, significance.

### Follow-up and events

Median follow-up was 5 years (2.6, 5) with a total of 2382 patient-years of observation time. Of the total population, 544 patients (88%) survived the first year. During follow-up, 72 (12%) patients were implanted with an LVAD, 145 (23%) patients received HTx, 31 (5%) patients received an LVAD prior to HTx, 186 (30%) received either transplant or LVAD implantation, and 178 (29%) patients died. Thirty-four patients (6%) died on either LVAD or after transplantation. In total, 330 (53%) experienced the combined outcome of either death, LVAD, or HTx. Among patients with LCOS, 45 (39%) died compared to 133 (26%) in the non-LCOS group. Furthermore, 48 (41%) patients with LCOS received advanced therapies compared to 138 (27%) in the non-LCOS group. Finally, the combined outcome of either LVAD, HTx, or death occurred in 84 (72%) patients with LCOS compared to 246 (49%) patients with non-LCOS.

### Prognosis and outcomes in ambulatory LCOS

#### All-cause mortality

**Figure 2:**
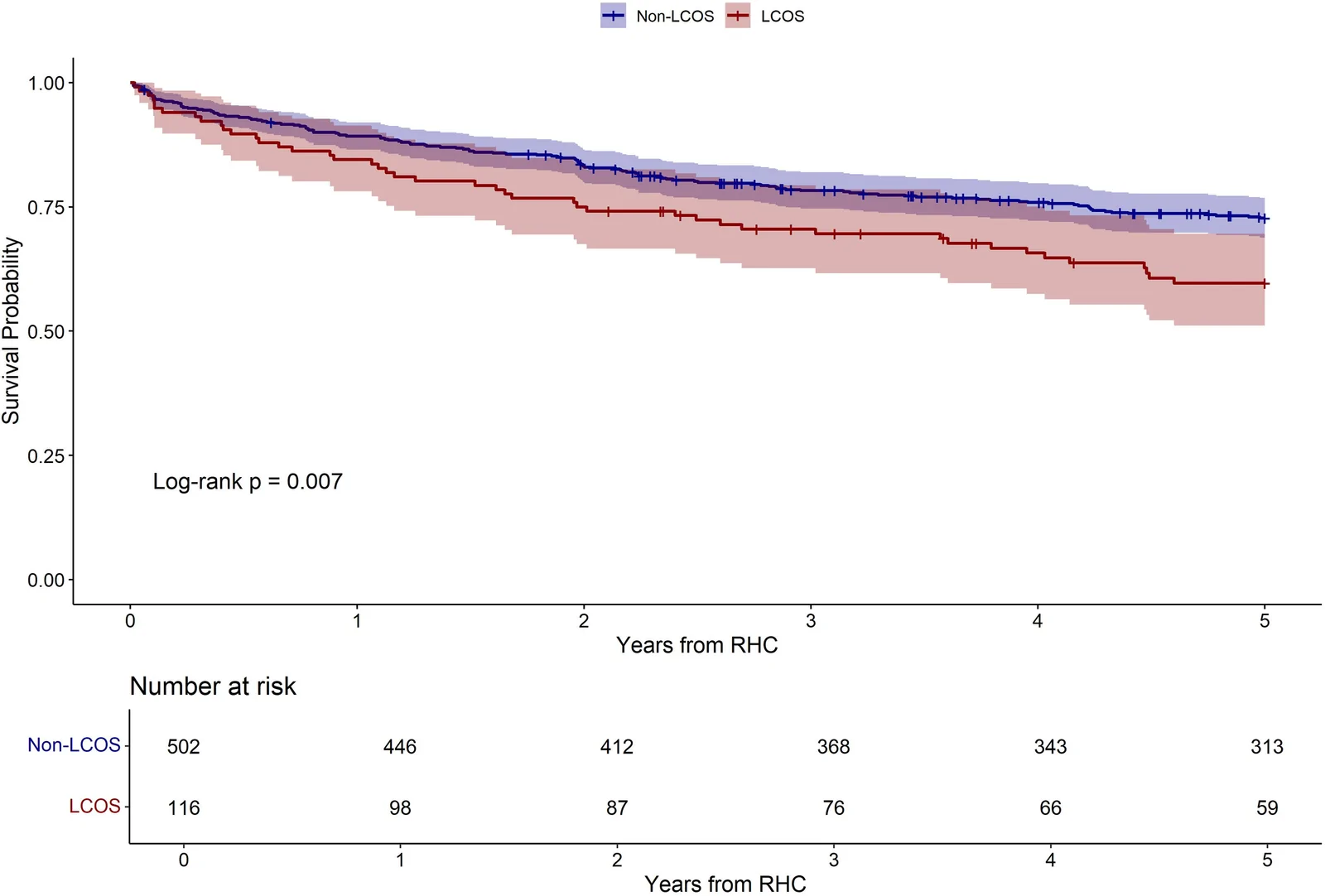
All-cause death by LCOS group

The survival curves for all-cause death demonstrate a worse prognosis in patients with LCOS (*Figure 2*). After five years, 60% and 73% were alive in the LCOS and non-LCOS group, respectively. The unadjusted hazard ratio (HR) of death for LCOS vs non-LCOS was 1.59 [95% CI 1.13 to 2.22], *P* = .008 which did not change notably after adjustment for age and sex. Although sex did not interact with the prognostic impact of LCOS status, we observed that continuous age did (*P* for interaction = .04). Age was strongly associated with mortality in both groups. However, the prognostic effect of LCOS varied across the age-spectrum and appeared not significantly associated with mortality in the youngest (<30 years) and oldest patients (>65 years). Patients with non-LCOS experienced a more rapid increase in hazard with increasing age compared to patients with LCOS.

In the adjusted model, we also included advanced therapy (time to earliest of LVAD or HTx) as a time-dependent covariate and found that patients with LCOS had a HR for death of 1.86 [95% CI 1.32 to 2.62], *P* = .0003. Thus, even after adjusting for utilization of advanced therapies, patients with LCOS had an 86% higher risk of death compared to patients with a relatively preserved cardiac index. Furthermore, we explored survival curves stratified in three cardiac index groups: <1.8, 1.8–2.2, and ≥2.2 L/min/m^2^ (Supplementary Figure S1) demonstrating a significant difference between the group with a normal cardiac index compared to the two lower cardiac index groups.

Continuous CVP, but not PCWP, interacted with LCOS status (*P* for interaction = .04). Thus, the presence of LCOS was significantly associated with mortality in the lower range of CVP between 1 and 12 mmHg, but lost its prognostic value when CVP exceeded 12 mmHg.

#### Advanced therapy

**Figure 3:**
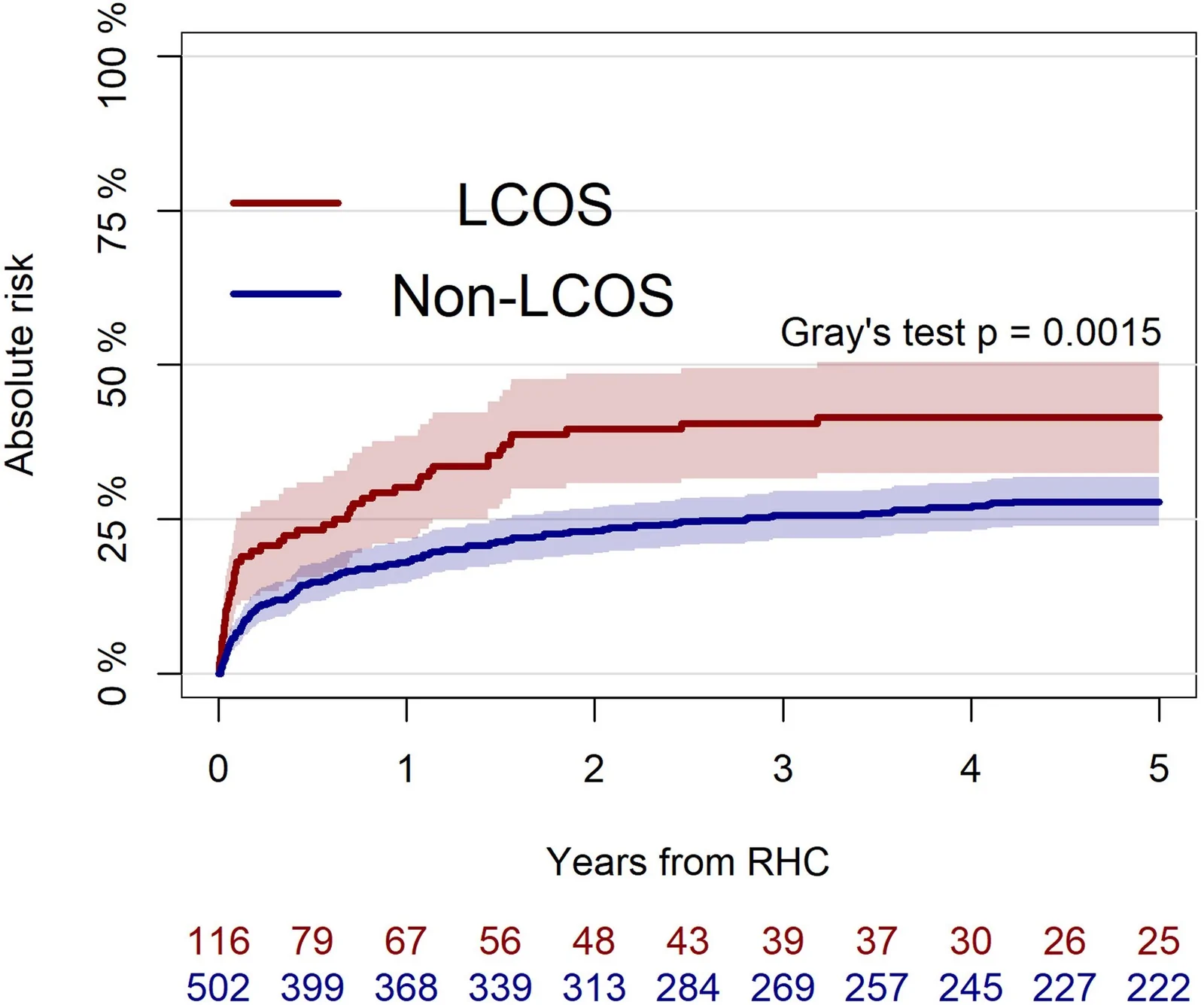
Cumulative incidence of advanced therapy (LVAD, HTx)

In a competing risk analysis, the cause-specific hazard in the LCOS vs non-LCOS group for advanced therapy was HR 1.84 [95% CI 1.33 to 2.56], *P* = .0003. Adjustments for age and sex did not change the result. The cumulative incidence plot of advanced therapy is displayed in *Figure 3*. As shown, when accounting for death as a competing risk, patients with LCOS have a significantly higher probability of advanced therapy. Thus, at 5 years, the chance of receiving advanced therapy was 41% [95% CI 33 to 50%] and 28% [95% CI 24 to 32%] in patients with LCOS and non-LCOS, respectively. The adjusted sub-distribution HR was 1.72 [95% CI 1.23 to 2.40], *P* = .002.

## Discussion

This study is the first to describe LCOS as well as its prognostic implications in an ambulatory population with HFrEF referred for invasive evaluation for advanced therapies. Several important findings were made (*Graphical Abstract*): First, ambulatory LCOS was found in almost a fifth of the population. Second, more than a quarter of patients with LCOS reported NYHA class II symptoms. Third, low SBP, high NT-proBNP and absence of RASi therapy were associated with LCOS. Fourth, while biomarkers of liver function did associate with LCOS, surprisingly estimated glomerular filtration did not. Finally, LCOS was strongly associated with increased mortality, irrespective of advanced therapies.

### Prevalence and detection of ambulatory LCOS

Limited data exist regarding the prevalence of ambulatory LCOS. A smaller study with data from a US population reported a prevalence of 40% using a less restrictive cardiac index cutoff of 2.2 L/min/m^2^.^11^ Applying this cutoff in our cohort would have resulted in a prevalence of LCOS of 42%. Thus, our data align with previous findings and further demonstrate that LCOS is frequently observed in carefully selected populations with suspected advanced HFrEF referred for invasive haemodynamic assessment. Furthermore, we identified risk markers of LCOS comparable to previously well-known indicators of advanced HF in general.^13^ Thus, the present study confirms that risk factors such as NYHA class III-IV, low blood pressure and LVEF, recent HFH, high NT-proBNP, and absence of RASi therapy can help identify patients with LCOS. However, more than a quarter of patients with LCOS were discordantly classified in NYHA class I-II, implying that reporting few symptoms does not rule out LCOS.

Regarding biochemical markers, we anticipated that renal function would associate with LCOS, since hypoperfusion is expected to cause end-organ dysfunction, but surprisingly eGFR was not associated with LCOS. Several factors may explain this finding. First, more patients in the non-LCOS group were RASi tolerant, which could lead to lower eGFR not representing intrinsic renal disease in this group. Second, as a significant proportion in both groups had reduced eGRF, it cannot be ruled out that evenly distributed noncardiac factors leading to a low eGFR were present in the two groups. Finally, renal adaption to the low output state may have played a role in the LCOS group. Indeed, prior studies have shown that eGFR correlates more closely with renal venous afterload than cardiac index.^14–16^ While further studies are needed to explain the lack of difference in eGFR in the two groups, it is clear from the current study that preserved eGFR does not rule out LCOS in ambulatory patients with HFrEF. Further, we observed that while abnormalities in common liver function tests were associated with LCOS, this signal disappeared for ALP and bilirubin when adjusting for CVP. However, ALT was persistently associated with LCOS despite adjustment for CVP, and so a doubling of ALT increased odds for LCOS by 25% (*Figure 1*). This is consistent with the assumption that reduced perfusion is associated with hypoxic liver injury, whereas ALP and bilirubin generally are more closely associated with liver congestion.

### Prognostic impact of LCOS and the importance of filling pressures

The results of the current study demonstrate that cardiac index is an independent predictor of outcomes, which has been a matter of debate in previous investigations.^17,18^ In this study, we adopted a conservative definition of LCOS (<1.8 L/min/m^2^). However, our data indicate that a prognostic impact of cardiac index is observed already at higher levels, i.e. 2.2 L/min/m^2^. While further studies are required to better define LCOS in ambulatory HF patients, a limit of 2.2 L/min/m^2^ in inotrope-independent patients has been previously proposed as a criterion for non-shock LCOS in HF and is consistent with the threshold used, for instance, for intervention with durable mechanical circulatory support in clinical trials.^4,19^ Furthermore, we observed that the prognostic value of LCOS varied with age and CVP (but not PCWP). Mechanisms behind these interactions are not completely clear. It could be speculated that end-organ function may be more tolerant to low cardiac index in younger patients and conversely less tolerant in patients >65 years. Especially in the elderly patients, comorbidities may play a relatively larger role in survival compared to central haemodynamics. Alternatively, this finding may also reflect that younger patients receive advanced therapies at an earlier stage of disease progression. The interaction with CVP may suggest that mortality in patients with lower CVP is primarily driven by forward failure, whereas in those with markedly elevated CVP it is more strongly influenced by congestion and right-sided failure, potentially attenuating the incremental adverse impact of cardiac index. Importantly, given the marginal statistical significance of these interactions, they may merely represent incidental findings and should therefore be interpreted with caution.

The need to characterize better the transition from stage C to D HF (‘ambulatory C2D’) is increasingly recognized since these patients are unlikely to benefit sufficiently from conventional GDMT.^20^ Part of the ambulatory C2D HF phenotype is related to hypoperfusion, but the extent to which hypoperfusion is present not just during (light) exercise but also at rest has not been well described yet. The current study adds important information about impairment of CO at rest in ambulatory HF patients and highlights that a significant proportion of these patients may indeed report NYHA II symptoms. A recent expert review highlights the importance of distinguishing between worsening HF and advanced HF.^21^ Our findings suggest that a considerable overlap exists between ambulatory patients with and without LCOS; notably, 21% of patients with LCOS had no prior HFH. Patients with ambulatory LCOS should likely be included in the C2D/advanced HF definition but also highlights the need for clinical markers beyond NYHA class to identify them and ensure proper risk assessment and consideration of advanced therapies also in this group. Specifically, our results indicate that RASi intolerance should weigh strongly in clinical decision-making, potentially enabling earlier referrals to advanced HF care centres.

### Strengths and limitations

This study includes granular data from a large cohort of patients with HFrEF and invasively assessed CO. However, several limitations exist. Most importantly, this study retrospectively included selected patients referred for evaluation for advanced therapies at a single advanced HF centre. This is an important limitation to the external validity, reducing direct generalizability to more general HFrEF populations. Second, haemodynamic measurements were obtained at a single point in time during rest and do not reflect dynamic changes in CO during exertion or longitudinally over time. Third, data were missing for some variables, but missingness was considered random. Lastly, routine measurement of lactate levels was not performed or recorded, limiting the ability to distinguish between compensated and uncompensated hypoperfusion.

## Conclusion

Low cardiac output state is common in ambulatory patients with HFrEF referred for invasive evaluation for advanced therapies. Normal liver and renal function do not rule out LCOS, but the absence of RASi therapy and recent HFH are among important markers of LCOS. Low cardiac output state is highly predictive of mortality, irrespective of left-sided filling pressure and advanced therapy.

## Supplementary Material

xvag215_Supplementary_Data

## Declarations

### Data Availability

Data is available from the corresponding author upon reasonable request.

### Pre-registered Clinical Trial Number

None supplied.

## References

[1] Crespo-Leiro MG, Metra M, Lund LH, Milicic D, Costanzo MR, Filippatos G, et al Advanced heart failure: a position statement of the Heart Failure Association of the European Society of Cardiology. Eur J Heart Fail 2018;20:1505–35. 10.1002/ejhf.1236 29806100

[2] Heidenreich PA, Bozkurt B, Aguilar D, Allen LA, Byun JJ, Colvin MM, et al 2022 AHA/ACC/HFSA guideline for the management of heart failure: a report of the American College of Cardiology/American Heart Association Joint Committee on Clinical Practice Guidelines. Circulation 2022;145:e895–1032. 10.1161/CIR.0000000000001063 35363499

[3] Chioncel O, Mebazaa A, Harjola VP, Coats AJ, Piepoli MF, Crespo-Leiro MG, et al Clinical phenotypes and outcome of patients hospitalized for acute heart failure: the ESC Heart Failure Long-Term Registry. Eur J Heart Fail 2017;19:1242–54. 10.1002/ejhf.890 28463462

[4] Verbrugge FH, Guazzi M, Testani JM, Borlaug BA. Altered hemodynamics and end-organ damage in heart failure: impact on the lung and kidney. Circulation 2020;142:998–1012. 10.1161/CIRCULATIONAHA.119.045409 32897746 PMC7482031

[5] Shah MR, Hasselblad V, Stinnett SS, Gheorghiade M, Swedberg K, Califf RM, et al Hemodynamic profiles of advanced heart failure: association with clinical characteristics and long-term outcomes. J Card Fail 2001;7:105–13. 10.1054/jcaf.2001.24131 11420761

[6] Reyentovich A, Barghash MH, Hochman JS. Management of refractory cardiogenic shock. Nat Rev Cardiol 2016;13:481–92. 10.1038/nrcardio.2016.96 27356877

[7] Naidu SS, Baran DA, Jentzer JC, Hollenberg SM, van Diepen S, Basir MB, et al SCAI SHOCK stage classification expert consensus update: a review and incorporation of validation studies: this statement was endorsed by the American College of Cardiology (ACC), American College of Emergency Physicians (ACEP), American Heart Association. J Am Coll Cardiol 2022;79:933–46. 10.1016/j.jacc.2022.01.018 35115207

[8] Baran DA, Grines CL, Bailey S, Burkhoff D, Hall SA, Henry TD, et al SCAI clinical expert consensus statement on the classification of cardiogenic shock: this document was endorsed by the American College of Cardiology (ACC), the American Heart Association (AHA), the Society of Critical Care Medicine (SCCM), and the society of thoracic surgeons (STS) in April 2019. Catheter Cardiovasc Interv 2019;94:29–37. 10.1002/ccd.28329 31104355

[9] Hochman JS, Sleeper LA, Webb JG, Sanborn TA, White HD, Talley JD;, et al Early revascularization in acute myocardial infarction complicated by cardiogenic shock. SHOCK investigators. Should we emergently revascularize occluded coronaries for cardiogenic shock. N Engl J Med 1999;341:625–34. 10.1056/NEJM199908263410901 10460813

[10] Mathew R, Di Santo P, Jung RG, Marbach JA, Hutson J, Simard T, et al Milrinone as compared with dobutamine in the treatment of cardiogenic shock. N Engl J Med 2021;385:516–25. 10.1056/nejmoa2026845 34347952

[11] Mullens W, Abrahams Z, Skouri HN, Taylor DO, Starling RC, Francis GS, et al Prognostic evaluation of ambulatory patients with advanced heart failure. Am J Cardiol 2008;101:1297–302. 10.1016/j.amjcard.2007.12.031 18435961

[12] Hansen BL, Deis T, Larsson JE, Ersbøll M, Rossing K, Schou M, et al Influence of obesity on invasive hemodynamics and prognosis in patients with heart failure. JACC Heart Fail 2025;13:725–36. 10.1016/j.jchf.2024.12.009 40117394

[13] Baumwol J . I Need Help”-A mnemonic to aid timely referral in advanced heart failure. J Heart Lung Transplant 2017;36:593–4. 10.1016/j.healun.2017.02.010 28258792

[14] Damman K, van Deursen VM, Navis G, Voors AA, van Veldhuisen DJ, Hillege HL. Increased central venous pressure is associated with impaired renal function and mortality in a broad spectrum of patients with cardiovascular disease. J Am Coll Cardiol 2009;53:582–8. 10.1016/j.jacc.2008.08.080 19215832

[15] Mullens W, Abrahams Z, Francis GS, Sokos G, Taylor DO, Starling RC, et al Importance of venous congestion for worsening of renal function in advanced decompensated heart failure. J Am Coll Cardiol 2009;53:589–96. 10.1016/j.jacc.2008.05.068 19215833 PMC2856960

[16] Hanberg JS, Sury K, Wilson FP, Brisco MA, Ahmad T, Ter Maaten JM, et al Reduced cardiac index is not the dominant driver of renal dysfunction in heart failure. J Am Coll Cardiol 2016;67:2199–208. 10.1016/j.jacc.2016.02.058 27173030 PMC4867078

[17] Aalders M, Kok W. Comparison of hemodynamic factors predicting prognosis in heart failure: a systematic review. J Clin Med 2019;8:1757. 10.3390/jcm8101757 31652650 PMC6832156

[18] Drazner MH . Refining the cold profile in patients with acute heart failure. Circ Heart Fail 2021;14:E008298. 10.1161/CIRCHEARTFAILURE.121.008298 33677979

[19] Mehra MR, Uriel N, Naka Y, Cleveland JC, Yuzefpolskaya M, Salerno CT, et al A fully magnetically levitated left ventricular assist device—final report. N Engl J Med 2019;380:1618–27. 10.1056/nejmoa1900486 30883052

[20] Dunlay SM, Pinney SP, Lala A, Stewart GC, McIlvennan C, Wong RP, et al Recognition of the large ambulatory C2D stage of advanced heart failure-A call to action. JAMA Cardiol 2025;10:391–8. 10.1001/jamacardio.2024.5328 39908057

[21] Palazzuoli A, Del Buono MG, La Vecchia G, Greene SJ, Ambrosy AP, Chioncel O, et al Worsening versus advanced heart failure: management and challenges. ESC Heart Fail 2025;12:3856–68. 10.1002/ehf2.15437 41045229 PMC12719834

