# Supplementary material for: Ambulatory low cardiac output state in heart failure: characteristics and outcomes
Source: ESC Heart Fail. 2026 Aug 19;13(4):xvag215. doi: 10.1093/eschf/xvag215 (PMC13505855; doi:10.1093/eschf/xvag215)
Supplement: Supplementary file 1 [file xvag215_supplementary_data.docx]

**SUPLEMENTARY MATERIAL**

**Supplemental Figure 1**. All-cause death stratified by cardiac index groups. CI = cardiac index, L/min/m^2^.
